# Expanding clinical phenotype in *CACNA1C* related disorders: familial mesial temporal lobe epilepsy

**DOI:** 10.1186/s42494-025-00231-5

**Published:** 2025-11-03

**Authors:** Chengzhe Wang, Xintong Guo, Yue Liu, Dingju Long, Heyu Zhang, Sijing Yin, Yinchao Li, Yicong Liu, Guanzhong Ni, Ziyi Chen

**Affiliations:** 1 Department of Neurology, Guangdong Provincial Key Laboratory of Diagnosis and Treatment of Major Neurological Diseases Department and Key Discipline of Neurology, The First Affiliated Hospital, Sun Yat-Sen University, National Key Clinical, No.58 Zhongshan Road 2, Guangzhou, 510080 China; 2 Department of Neurology, The Seventh Affiliated Hospital, Sun Yat-Sen University, Shenzhen, 518107 China

**Keywords:** Familial mesial temporal lobe epilepsy, *CACNA1C* gene, Whole-exome sequencing, Genotype–phenotype correlation

## Abstract

**Background:**

To provide new insights into the pathological mechanisms of epilepsy associated with variants in the calcium channel voltage-dependent L-type alpha1C subunit gene (*CACNA1C*, NM_001129837) and to expand the phenotype of *CACNA1C*-associated neurological disorders: familial mesial temporal lobe epilepsy (FMTLE).

**Methods:**

We conducted a comprehensive analysis of clinical data from a family affected by FMTLE and carried out genetic screening of *CACNA1C* variants through whole-exome sequencing combined with Sanger sequencing for validation. The clinical characteristics of FMTLE were systematically reviewed, and the pathogenic potential of the identified variant was assessed following the guidelines established by the American College of Medical Genetics and Genomics (ACMG). To explore the underlying pathogenic mechanisms, we utilized bioinformatics tools alongside molecular dynamics simulation methods.

**Results:**

A novel *CACNA1C* variant (c.5480G > A, p.R1827Q) was identified in a large family with FMTLE. Unlike previous reports, the clinical phenotype of this genotype differs from previous reports, being mild, with focal to bilateral tonic–clonic seizures being more common. Bioinformatics analysis and molecular dynamics simulations indicated that this variant induces local structural changes in the protein.

**Conclusions:**

The findings of this study provide new insights into the complex molecular mechanisms underlying *CACNA1C* variants and their correlations with patient phenotypes. This research is the first to identify *CACNA1C* as a potentially new pathogenic gene in FMTLE.

**Supplementary Information:**

The online version contains supplementary material available at 10.1186/s42494-025-00231-5.

## Background

The calcium channel voltage-dependent, L type, alpha1C subunit gene (*CACNA1C*) is located on the short arm of chromosome 12 (12p13.3) and encodes the Cav1.2 protein. The *CACNA1C *channel comprises four homologous domains (DI-DIV), each consisting of six transmembrane segments, cytoplasmic segments, and transmembrane linker segments [1, 2]. Cav1.2 is an evolutionarily conserved protein expressed in the heart, brain, lungs, and smooth muscles, and it plays a critical role in Ca^2^⁺ signaling, cellular and neuronal excitability, muscle contraction, and gene expression regulation [3, 4]. The *CACNA1C* gene variants were initially found to be associated with various cardiac arrhythmias, such as Timothy syndrome and Brugada syndrome. In recent years, the clinical phenotypes related to *CACNA1C *have been increasingly complex [5, 6], including schizophrenia, bipolar disorder, epilepsy, migraine, and ataxia. As more pathogenic mutations of *CACNA1C* are discovered, more epilepsy phenotypes caused by mutations in this gene have been reported. Previous case reports described a case of neonatal epileptic encephalopathy and identified a new missense variant in *CACNA1C *(c.4087G > A, p.V1363M) [7]. In another study [6], ten patients with non-truncating variants of *CACNA1C* gene were reported, and the patients exhibited a spectrum of epilepsy syndromes and phenotypes, ranging from medically refractory infantile epileptic encephalopathy to later onset focal or generalized seizures that responded well to anti-seizure medications.

Genetically related epilepsy constitutes one of the six primary etiologies of epilepsy. As of now, over a thousand genes associated with epilepsy have been recognized [8]. And hereditary epilepsy imposes an increased disease burden on the entire family of the afflicted individuals. Temporal lobe epilepsy (TLE) is the most common focal epilepsy in adulthood. Familial TLE includes two different subtypes: the lateral form, characterized as autosomal dominant epilepsy with auditory features (ADEAF) [9], and the more prevalent mesial subtype, known as familial mesial temporal lobe epilepsy (FMTLE) [10]. According to the 2022 ILAE classification and the definition of variable age epilepsy syndromes [11]. FMTLE is a type of focal epilepsy syndrome that arises from genetic, structural, or a combination of genetic and structural causes. In preceding reports, FMTLE seizures have customarily been reported as focal. A majority of the patients suffer from déjà vu, commonly accompanied by dream-like experiences, fear, panic, a feeling of slow motion, visual or auditory illusions, and autonomic symptoms. FMTLE is classified into two forms: (1) benign cases without hippocampal sclerosis (HS) or febrile seizures (FS), and (2) cases characterized by the presence of HS and/or FS [12]. To date, FMTLE has been linked to polygenic etiologies [13], yet the identification of causative variants for FMTLE remains elusive, with only a few reports have pinpointed such variants in FMTLE. Beyond a limited number of dominant pedigrees harboring pathogenic mutations within the *GATOR1* complex genes (*DEPDC5, NPRL2, and NPRL3*), conclusive findings regarding monogenic contributions to FMTLE remain lacking [14–16]. Moreover, in our previous research, we also reported two FMTLE families with *LGI1 *variants [17]. To the best of our knowledge, there have been no reports on *CACNA1C* single-gene variants causing FMTLE.

This study aims to expand the phenotype associated with *CACNA1C* variants and utilize bioinformatics tools to explore whether *CACNA1C* is the pathogenic gene of familial temporal lobe epilepsy.

## Materials and methods

### Participants

This research received formal approval from the Ethics Committee. We carried out a comprehensive review of patients who were hospitalized at the First Affiliated Hospital of Sun Yat-sen University between January 1, 2019, and May 31, 2023. A total of three families were identified as having FMTLE, encompassing 145 members, among whom 15 were confirmed to be affected. Genetic analysis revealed that one of these families carried a mutation in the *CACNA1C* gene.

### Inclusion criteria and exclusion criteria

Based on the 2017 International League Against Epilepsy (ILAE) operational classification of seizure types [18] and the 2022 ILAE classification and definitions of epilepsy syndromes with variable age of onset [11], families diagnosed with FMTLE and found to have *CACNA1C* variants through whole-exome sequencing (WES) were included in this study. Exclusion criteria encompassed patients whose FMTLE was linked to other pathogenic genes or those with epilepsy attributed to alternative, well-defined etiologies.

### Methods

Medical histories of FMTLE patients harboring variations in the *CACNA1C *gene were compiled, including video electroencephalogram (VEEG) recordings and 3.0 T brain magnetic resonance imaging (MRI) scans. The categorizations of "Drug responsive" and "Undefined" in drug responsiveness were derived from the "Definition of drug resistant epilepsy: Consensus proposal by the ad hoc Task Force of the ILAE Commission on Therapeutic Strategies" [19]. The WES and Sanger verification outcomes for these families were obtained from the Guangzhou Forevergen Medical Laboratory. Comprehensive methodologies detailing the exome analysis, Sanger sequencing process used in this study, as well as the systematic approach to variant identification and selection of the *CACNA1C* allele, are described in Supplementary Methods S1 and S2. Eight species representing diverse evolutionary levels were randomly selected as research subjects: Homo sapiens, Mus musculus, Rattus norvegicus, Bos taurus, Cavia porcellus, Equus caballus, Pongo abelii, and Danio rerio. The sequences of these species were retrieved from the NCBI database (https://www.ncbi.nlm.nih.gov). Comparative analysis of the aforementioned sequences was conducted using the MUSCLE method integrated within the MEGA X software to investigate whether the amino acid sites involved in this study exhibit evolutionary conservation [20]. We also assessed the conservation of this locus by aligning the data using the UCSC database (https://genome.ucsc.edu). To analyze the pathogenicity of the variant sites, clinical data were integrated with various online pathogenicity analysis tools, including the VarCards database (http://varcards.biols.ac.cn) and guidelines from the American College of Medical Genetics and Genomics (ACMG) [21]. The NCBI database was used to search for structural domain information of the Homo sapiens CACNA1C (NM_001129837). Owing to the substantial molecular weight of the CACNA1C protein, we utilized AlphaFold and trRosetta to conduct three-dimensional modeling of CACNA1C [22, 23]. Moreover, we implemented the protein preparation wizard module in Schrodinger software to process the obtained protein crystals, encompassing protein preprocessing and protein energy minimization. Building upon the wild-type modeling, we employed PyMOL software to perform manual mutations [24] to compare the protein structures before and after the *CACNA1C *p.R1827Q. The Gromacs 2022.3 software was used for the molecular dynamics simulation (Supplementary Methods S3) [25, 26].

## Results

### Clinical data

**Fig. 1:**
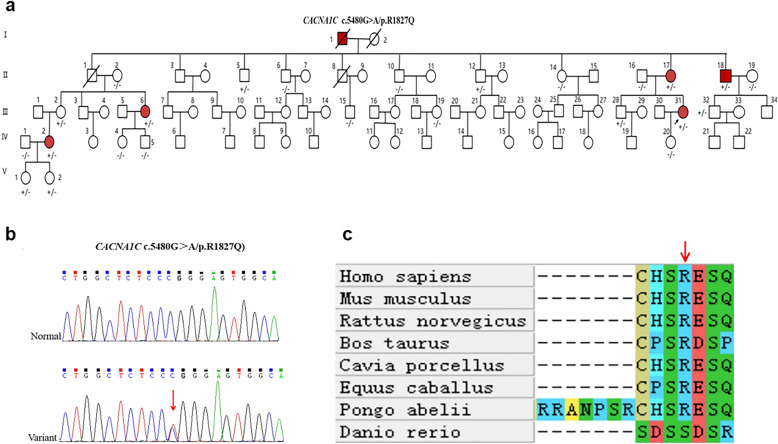
Clinical features of the family. **a** Pedigree chart. ± : Patients carrying the p.R1827Q variant; -/-: Patients not carrying the p.R1827Q. Red indicates patients with epilepsy seizures. **b** Sanger sequencing confirmed the *CACNA1C* variants (c.5480G > A, p.R1827Q). **c** Conservation analysis of residue p.R1827Q of *CACNA1C* across eight species

**Fig. 2:**
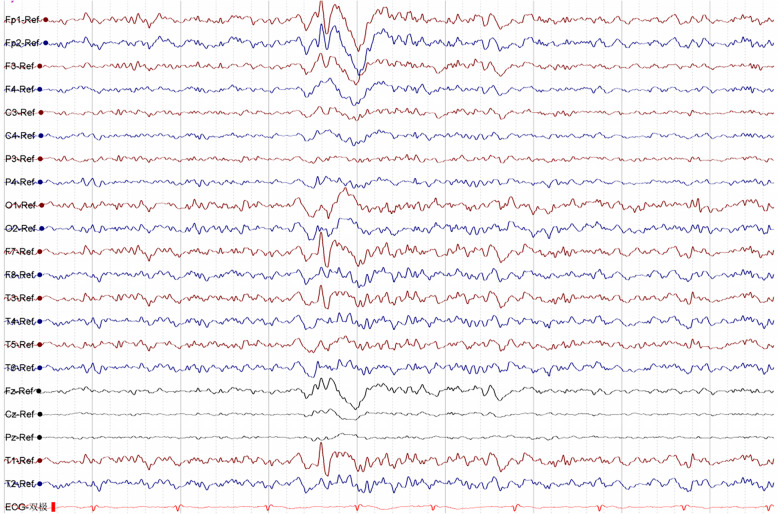
The proband’s (III-31) 24 h VEEG indicating an interictal left temporal epileptiform abnormality, and the brain MRI revealing no discernible abnormalities

The family consists of 79 members in total, of which six suffer from epileptic seizures (I-1 has passed away), and their clinical characteristics are summarised in Table 1. All patients and their relatives have denied having cardiovascular disease, hearing disorders, focal cognitive seizures, sensory aphasia seizures, or a history of febrile convulsions. we retrospectively analyzed available clinical histories and family interview information, including the living environment of family members and potential common environmental exposure factors, which could potentially influence the occurrence and development of epilepsy. However, after investigation, the living environment of all family members was found not to have an impact on the occurrence and development of epilepsy. The family tree and variants are shown in Fig. 1a. The proband (III-31), a 35-year-old right-handed woman, initially presented with marked fear sensations during sleep at the age of 29 years, which were subsequently developed tonic–clonic seizures. Within a year, analogous symptoms reemerged upon awakening. Following her second seizure episode, she sought medical attention at our institution and initiated therapy with lamotrigine (LTG), titrated up to a maximum daily dose of 175 mg. Since then, she has not experienced focal to bilateral tonic–clonic seizures again. Nevertheless, she continues to experience focal aware seizures characterized by fear, which occur with a frequency of 2–6 episodes annually and are consistently triggered by pronounced fatigue. The 24 h VEEG revealed intermittent left temporal epileptiform abnormalities (Fig. 2), while the brain MRI did not indicate any significant MRI abnormalities or evidence of hippocampal sclerosis (HS). The proband’s mother (II-17), an elderly right-handed female aged 61, initially manifested pronounced fear sensations during wakefulness at the age of 30. These episodes were subsequently followed by tonic–clonic epileptic seizures, which have recurred at an annual frequency of 1–3 times. Her clinical presentation is consistent with a history of focal epilepsy, as evidenced by the intermittent nature of her symptoms and the absence of other neurological deficits on examination. She also experienced pronounced fear sensations, which also occurred independently, at a frequency of 1–3 times per month. Starting at the age of 53, she began taking 1000 mg of valproic acid (VPA) daily, which effectively controlled her symptoms. However, without consulting an epilepsy specialist, she independently reduced the dosage to 500 mg/day, and since then, she has not experienced any seizures since. The proband's uncle (II-18), a 60-year-old right-handed man, presented with his initial focal to bilateral tonic–clonic seizure at the age of 32. His clinical presentation is similar to that of the proband. Despite initiating VPA therapy at a dose of 1000 mg/day in a non-regular manner following the onset of symptoms, his epilepsy remained refractory to treatment, with inadequate control of seizure frequency and severity. III-6 is a 47-year-old right-handed woman, the cousin of the proband, experiencing her initial onset at age 33. She presents with clinical symptoms characterized by fear and focal to bilateral tonic–clonic seizures. Initiation of regular VPA therapy at 500 mg/day began at age 43, and she has been free of the aforementioned symptoms since commencing treatment. IV-2, a 33-year-old right-handed woman and the niece of the proband, had her first seizure at age 24. The type of seizures she experiences is consistent with those of the proband. Since the onset of her condition, IV-2 has not utilized any anti-seizure medications (ASMs), resulting in the persistence of her symptoms to the present day.

**Table 1:** Clinical and genetic characteristics of epilepsy patients in family

Member	Variant	Gender	Age	Age of seizure onset	Disease course	Seizure type	Seizure frequency	VEEG (Interictal)	MRI	ASMs	Drug responsiveness
II-17	c.5480G > A p. R1827Q	Female	61yr	30 yr	31 yr	Fear	1–3/m	NA	NA	VPA (500 mg/d)	Drug responsive
						Focal to bilateral tonic–clonic	1–3/yr				
II-18	c.5480G > A p. R1827Q	Male	60 yr	32 yr	28 yr	Fear	3–5/m	NA	NA	VPA (1000 mg/d)	Undefined
						Focal to bilateral tonic–clonic	3–5/yr				
III-6	c.5480G > A p. R1827Q	Female	47 yr	33 yr	14 yr	Fear	1–2/m	NA	NA	VPA (500 mg/d)	Drug responsive
						Focal to bilateral tonic–clonic	1–2/yr				
III-31	c.5480G > A p. R1827Q	Female	35 yr	29 yr	6 yr	Fear	2–6/yr	Left temporal epileptiform abnormality	N	LTG (175 mg/d)	Undefined
						Focal to bilateral tonic–clonic	2/6 yr				
IV-2	c.5480G > A p. R1827Q	Female	33 yr	24 yr	9 yr	Fear	1–2/m	NA	NA	NA	Undefined
						Focal to bilateral tonic–clonic	1–3/yr				

*ASMs* Anti-seizure medications, *LTG* Lamotrigine, *m* month, *MRI* Magnetic resonance imaging, *VEEG* Video electroencephalogram, *N* Normal, *NA* Not available, *VPA* Valproic acid, *yr* year

### Genetic analyses

Six individuals within the family manifest epilepsy (I-1 has passed away). WES was performed on four patients, alongside Sanger verification for twenty relatives. Furthermore, all subjects who underwent WES had previously undergone genetic variant screening associated with FMLTE. This comprehensive analysis ultimately identified the *CACNA1C* (c.5480G > A, p.R1827Q) as the sole shared variant (Fig. 1b). Among the four patients who underwent WES, three (II-17, III-6, III-31) harbored an identical *KCNT1* variant, with the proband (III-31) additionally carrying *CTP2* and *SCN1B* variants. A comprehensive list of potential candidate genes is provided in Table S1. This variant locus has not been previously reported before and exhibited incomplete penetrance in its genetic inheritance pattern. Multiple "harmful" predictive software programs suggest that this variant is likely pathogenic. According to the ACMG guidelines for the classification of sequence variants, the pathogenicity assessment of R1827Q indicated a classification of "uncertain significance" (Table S2). Conservation analysis of 8 species (human, Mus musculus, Rattus norvegicus, Bos taurus, Cavia porcellus, Equus caballus, Pongo abelii, Danio rerio) indicated that *CACNA1C* was relatively conserved site among mammals (Fig. 1c).

### Protein structure prediction

**Fig. 3:**
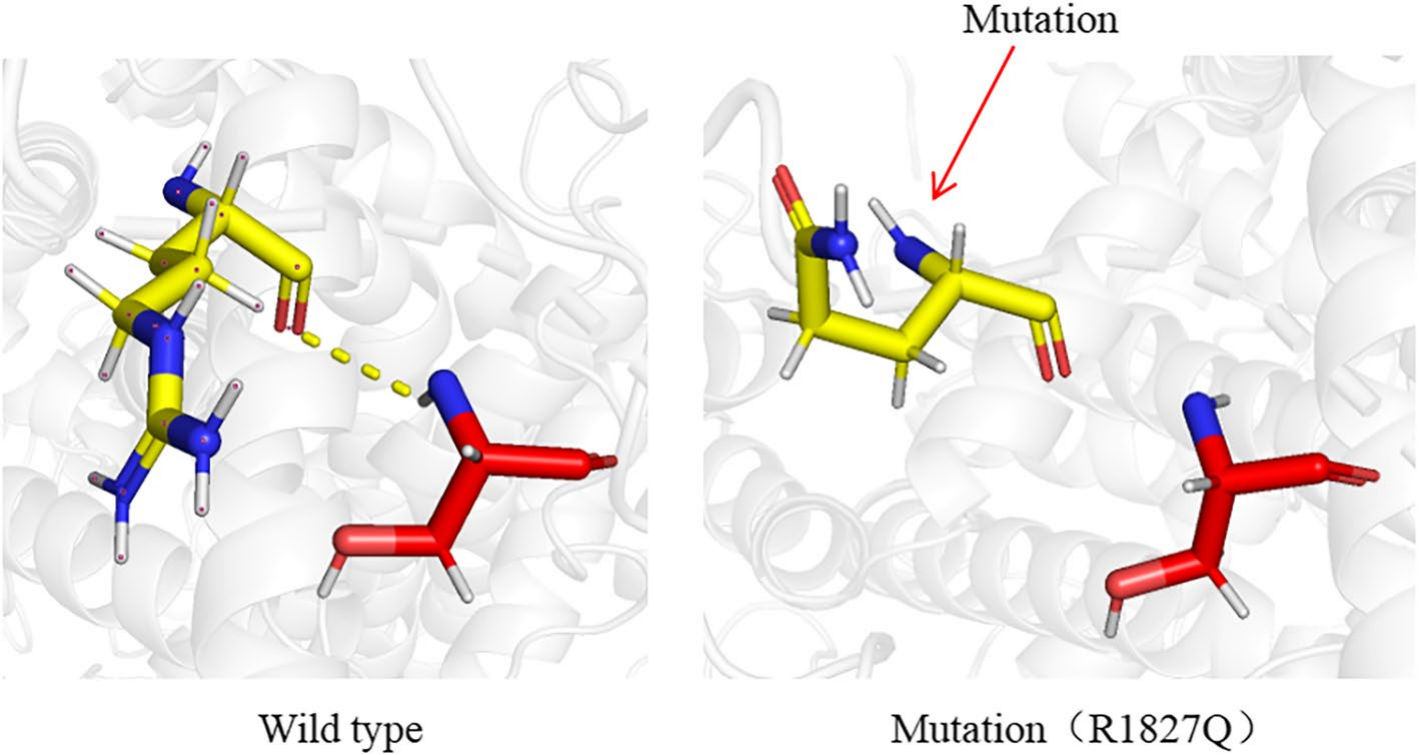
A 3D protein structure prediction of the variants. The results indicated that after the *CACNA1C* (c.5480G > A, p.R1827Q) variant, there was a reduction in hydrogen bonds

We utilized AlphaFold2 and trRosetta to perform crystal structure modeling of the CACNA1C protein. Based on data from the NCBI database, the R1827Q variant was situated in the voltage-gated calcium channel subunit alpha, C-term domain (CAC1F_C), which is the C-terminal region of voltage-gated calcium channel subunit alpha in higher eukaryotes (Table S3). Subsequently, we then employed PyMOL software to compare the CACNA1C protein structures preceding and following the variant site (Fig. 3). Our analysis revealed that the variant results in a decreased number of hydrogen bonds.

### Molecular dynamics simulation

In this simulation, we illustrated the root mean square deviation (RMSD) and root mean square fluctuations (RMSF) using a schematic comparison of the wild-type and mutant *CACNA1C* (c.5480G > A, p.R1827Q) proteins (Fig. S1–S2). The analysis revealed that although the overall patterns of RMSD and RMSF were comparable between the two protein forms, the mutant protein exhibited consistently lower RMSD values. Moreover, the RMSF values for the mutant specifically decreased within the 1800–1950 residue region. These findings suggest that the structural conformation of the mutant protein is more compact than that of the wild type.

The radii of gyration (Gyrate) and solvent-accessible surface area (SASA) were computed and visualized for both the CACNA1C wild-type (WT) and mutant (MT) groups (Fig. S3–S4). Analysis reveals that the Gyrate and SASA values for the MT group tend to be reduced compared to those of the WT group. This indicates that the folding of the mutated protein structure is more compact and has a smaller surface area.

Furthermore, we analyzed the variations in the number of hydrogen bonds (H-bonds) in both the *CACNA1C* WT and MT groups (Fig. S5). The results show a slight reduction in the number of hydrogen bonds within the protein following the *CACNA1C* (c.5480G > A, p.R1827Q) mutation. These findings suggest that local structural changes have occurred within the protein in the MT group.

## Discussion

Previous reports have demonstrated that FMTLE cases are more common during adolescence and adulthood, with a female preponderance [13, 27]. The epileptic seizures characteristic of FMTLE are predominantly focal, often accompanied by déjà vu, and these seizures rarely progress to bilateral tonic–clonic seizures [13]. The EEG of patients with FMTLE may be normal, or exhibit mild temporal slow waves and interictal temporal epileptiform abnormalities [13, 28]. Typically, patients with FMTLE show no obvious abnormalities on brain MRI [13]. A total of five patients were enrolled in this study. All experienced epileptic seizures during adulthood, and the type and manifestation of seizures were highly similar among each case. Notably, throughout the course of the disease, all patients experienced focal to bilateral tonic–clonic seizures, which contrasts with previously reported cases of FMTLE. This phenomenon was also observed in two additional FMTLE families reported by us, suggesting that this type of seizure was also a common type in FMTLE [17]. In our study, only the proband among the five patients completed a 24 h VEEG and brain MRI. Regrettably, due to feelings of shame about their condition, the other patients have refused to undergo MRI and VEEG examinations. Currently, the primary treatment approach for FMTLE is the control of epileptic seizures, with the majority of patients experience seizures control through initial medication. In this study, apart from the proband, other patients visited the local hospital after the onset of the disease and chose VPA as the anti-seizure medication. Two patients who took the medication regularly had a good response to VPA. This was consistent with our previous two FMTLE cases. As mentioned before, we attribute this to the mild and drug-responsive clinical phenotype of FMTLE [17].

In this study, a family was diagnosed with FMTLE. WES identified multiple several genetic variants, including *CACNA1C*. However, due to inconsistent inheritance patterns among the affected members, these variants are unlikely to be causative. Notably, the *CACNA1C* (c.5480G > A, p.R1827Q) variant has not previously been associated with FMTLE. Seven family members carrying this variant did not exhibit epilepsy. Incomplete dominant inheritance or mild, overlooked symptoms could explain these findings. This study identified significant variations in the clinical presentation of this genotype, differing from previous reports, characterized by a milder phenotype. Focal to bilateral tonic–clonic seizures were observed more often in these cases. In terms of genetic factors, the frequency of FMTLE in relatives of affected individuals is lower than predicted by the Mendelian dominant inheritance model, and only a few families follow recessive inheritance patterns. Therefore, FMTLE is considered a complex genetic disorder that may involve multiple genes or factors. Current research suggests that FMTLE is associated with multigenic causes, but a few reports have identified potential pathogenic variants, such as dominant families carrying pathogenic mutations in the *GATOR1* complex genes (*DEPDC5*, *NPRL2*, and *NPRL3*). This study, along with previous research, provides preliminary evidence for the genetic basis and pathogenic mechanisms of FMTLE, and opens new avenues for future studies.

In the brain, Cav1.2 is predominantly expressed in the hippocampus, thalamus, cerebral cortex, suprachiasmatic nucleus, and cerebellum [29–31]. Previous research have demonstrated that pathogenic variants of the *CACNA1C* gene are historically associated with cardiac arrhythmias, particularly long QT syndrome, Brugada syndrome, and Timothy syndrome. Additionally, *CACNA1C *variants have been linked to psychiatric disorders such as schizophrenia, bipolar disorder, and major depressive disorder [32], as well as a range of neurological conditions including ataxia, epilepsy, intellectual disability, and hypotonia [6]. Previous studies have shown that pathogenic mutations in the *CACNA1C *gene are closely associated with various epilepsy phenotypes, including neonatal-onset epileptic encephalopathy (NOEE), generalized epilepsy, and focal epilepsy, with some cases exhibiting fever sensitivity [6, 7]. Additionally, these mutations are more commonly observed in populations with non-truncating variants. A recent study found that abnormal expression of the α1c subunit in the hippocampal tissue of patients with temporal lobe epilepsy (TLE) suggests that *CACNA1C *may be involved in the pathogenesis of TLE [33]. However, no literature has yet reported the association between *CACNA1C* variant and FMTLE. The variant identified in this study differs significantly from previously reported *CACNA1C*-related epilepsy mutations, with a milder clinical presentation and typical focal seizures associated with medial temporal lobe involvement.

We believe that the broad clinical heterogeneity related to CACNA1C may be closely associated with its unique genetic characteristics. The gene undergoes alternative splicing and RNA editing, producing more than 200 distinct transcripts, which encode L-type calcium channels with varying functions and kinetics [34]. The regional differences in the abundance of CACNA1C transcripts across different brain regions may be the cause of its neural phenotypic heterogeneity [35]. Studies have shown that *CACNA1C *variant can lead to loss of function, neutral effects, or gain of function, thereby altering calcium ion influx and triggering seizures. Patch clamp experiments have confirmed this [6]. In epilepsy syndromes, most mutations result in loss of function, although the precise mechanisms remain unclear. Rodan and colleagues found that de novo non-truncating mutations associated with intellectual disability and epilepsy may have their neurological severity linked to disruption of the excitation-inhibition balance. Both increases and decreases in calcium conductance can provoke seizures and impair long-term potentiation, which explains why missense mutations, which alter channel conductance, tend to exhibit dominant-negative effects, while truncating mutations leading to haploinsufficiency are more tolerable [6]. Additionally, CACNA1C channels may also be expressed in GABAergic inhibitory interneurons, and their pathogenic mechanisms are similar to the loss-of-function mutations in *SCN1A* seen in Dravet syndrome [36]. Han et al. demonstrated that L-type calcium channel blockers can enhance the function of γ-aminobutyric acid A (GABA_A_) receptors, partially alleviating the occurrence of seizures [37]. Moreover, we observed inconsistent inheritance patterns of this mutation within the family, with seven family members carrying the variant but not exhibiting any seizure symptoms. This may be explained by incomplete dominance inheritance or the presence of mild, unnoticed symptoms. In conclusion, the CACNA1C gene mutation identified in this study differs from existing research in terms of both clinical features and inheritance patterns, suggesting that this mutation may exhibit different pathogenic effects in various clinical contexts. Future studies should further explore the specific mechanisms of this mutation and analyze its phenotypic differences in FMTLE and other epilepsy subtypes using larger sample sizes.

This study assessed the pathogenicity of the variant, concluding that the pathogenicity of the variant is classified as "uncertain significance". Although the ACMG guidelines for pathogenicity assessment are widely applied in clinical genetics, they have notable limitations. 1. Reliance on existing knowledge may lead to inaccuracies when analyzing newly discovered genes or mutations. 2. Genetic heterogeneity and phenotypic variability may complicate the prediction of individual phenotypes and pathogenicity. 3. The functional of unknown mutations are often insufficiently addressed. Given the limited reports associating *CACNA1C* mutations with epilepsy, while predictive tools and ACMG assessments provide valuable insights, they may not provide a definitive evaluation of pathogenicity. The specific mutation results in the substitution of arginine (Arg, R) with glutamine (Gln, Q) at position 1827. Arginine at position R1827 is a positively charged amino acid that plays a critical role in the interaction between proteins and negatively charged molecules. This mutation occurs within the CAC1F_C domain of the protein, a region located at the C-terminal region of the voltage-gated calcium channel subunit alpha in higher eukaryotes. Substitution of arginine with the neutral amino acid glutamine may alter the conformation of the CAC1F_C domain or disrupt its interactions with other proteins, potentially impairing the function of the calcium channel. In this study, this mutation resulted in a slight reduction in the number of local hydrogen bonds, which may affect the key functions of the protein. Molecular dynamics simulations indicate that, compared to the wild-type protein, the p.R1827Q mutant exhibits a more compact structure, reduced dynamic flexibility, and decreased surface exposure, while the number of internal hydrogen bonds in the local structure is also reduced. Specifically, although the overall structures of the WT and MT proteins are similar, in the local region—particularly between residues 1800 and 1950—the mutation leads to structural densification and diminished dynamic fluctuations. Changes in RMSD demonstrate that the mutation causes a shift in protein conformation, whereas the decrease in RMSF reflects reduced flexibility in this region. Since voltage-gated calcium channels rely on dynamic conformational changes to achieve ion gating, the reduction in local flexibility will affect the conformational rearrangements required during channel activation and deactivation, thereby impairing normal calcium ion conduction. Furthermore, the mutant protein exhibits a more compact structure than the wild-type, as evidenced by a reduced radius of gyration and lower SASA values. This structural densification may limit the interactions between the protein and regulatory proteins or ligands and reduce the efficiency of contact with essential cofactors, thereby affecting the conformational regulation and functional performance of the calcium channel. The p.R1827Q mutation replaces the positively charged arginine with neutral glutamine, disrupting the original charge balance and perturbing the salt bridges and hydrogen bond network that maintain protein stability. The reduction in hydrogen bonds indicates a decline in local structural stability, increasing the risk of protein misfolding or degradation, which in turn leads to abnormal calcium channel function. In summary, the normal function of calcium channels depends on the dynamic conformational changes of the protein, which are critical for regulating calcium ion flow. The aforementioned structural changes will disrupt the normal gating mechanism of the calcium channel, leading to abnormal calcium ion flow and ultimately triggering epilepsy. Therefore, based on the physiological and pathological mechanisms of *CACNA1C* in the central nervous system, we hypothesized that variations in *CACNA1C* may be associated with mesial temporal lobe epilepsy.

Earlier investigations have highlighted the rarity of monogenic causes in FMTLE, pointing instead to a likely polygenic origin [38]. However, unlike our current analysis, which incorporated WES alongside Sanger sequencing, the cited studies relied on genome-wide association studies (GWAS). Consequently, we cannot entirely rule out the possibility that the familial case we observed may have been driven by a single-gene inheritance mechanism.

## Conclusions

Our findings suggest that *CACNA1C* may represent a novel pathogenic gene associated with FMTLE. Future studies should aim to validate the pathogenic role of *CACNA1C* in FMTLE through cellular and animal experiments and to elucidate its specific pathological mechanisms.

## Supplementary Information

Supplementary Material 1.

## Data Availability

The data that support the findings of this study are available from the corresponding author upon reasonable request.

## Abbreviations

ACMG: American College of Medical Genetics and Genomics
ADEAF: Autosomal dominant epilepsy with auditory features
Arg, R: Arginine
ASM: Anti-seizure medications
CACNA1C: Calcium Channel Voltage-dependent L-Type Alpha 1C
CAC1F_C: Calcium Channel Subunit Alpha, C-term domain
EAF: Epilepsy with auditory features
FMTLE: Familial mesial temporal lobe epilepsy
FS: Febrile seizures
GABA A: Gamma-aminobutyric acid type A
Gln, Q: Glutamine
GWAS: Genome-wide association studies
H-bonds: Hydrogen bonds
HS: Hippocampal sclerosis
ILAE: International League Against Epilepsy
LTG: Lamotrigine
MRI: Magnetic resonance imaging
MT: Mutant
RMSD: Root mean square deviation
RMSF: Root mean square fluctuations
SASA: Solvent-accessible surface area
TLE: Temporal Lobe Epilepsy
VEEG: Video electroencephalogram
VPA: Valproic acid
WES: Whole-exome sequencing
WT: Wild-type
