# Supplementary material for: Expanding clinical phenotype in CACNA1C related disorders: familial mesial temporal lobe epilepsy
Source: Acta Epileptol. 2025 Nov 3;7:45. doi: 10.1186/s42494-025-00231-5 (PMC12581321; doi:10.1186/s42494-025-00231-5)
Supplement: Supplementary file 1 — Supplementary Material 1. [file 42494_2025_231_MOESM1_ESM.docx]

**Appendix. Supplementary materials**

**Contents**

**[Section 1: Supplementary Figures S1-S5](#_Toc151507331) 2**

**[Section 2: Supplementary Tables S1-S3 4](#_Toc151507332)**

**[Section 3: Supplementary Methods S1-S3 7](#_Toc151507333)**

# Section 1: Supplementary Figures S1-S5


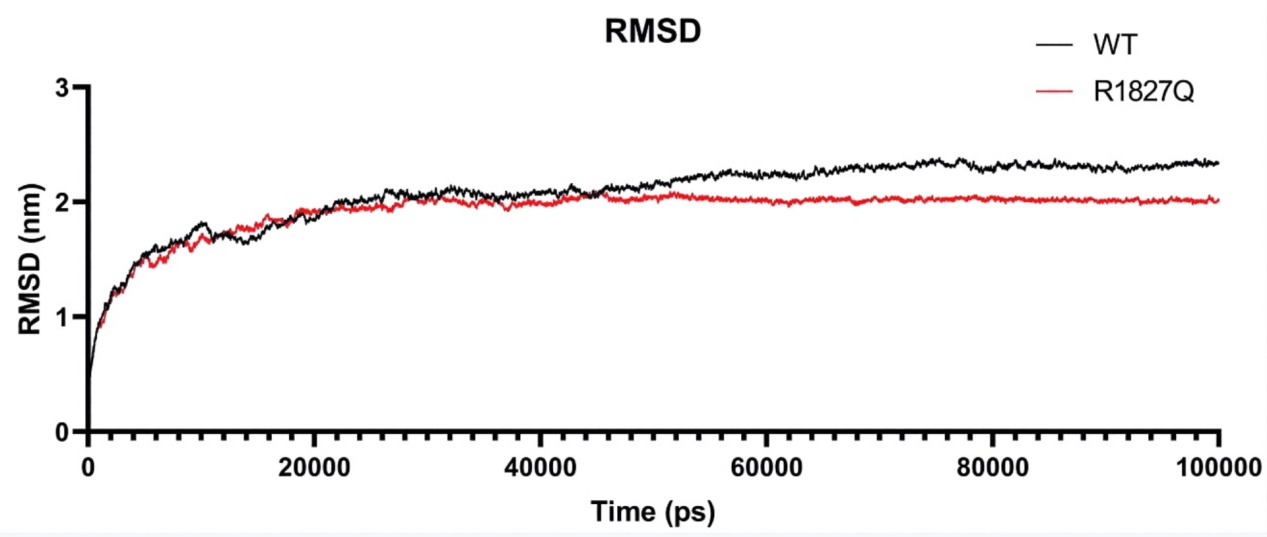


**Fig. S1**. Comparison of *CACNA1C* RMSD before and after *CACNA1C* p.R1827Q.


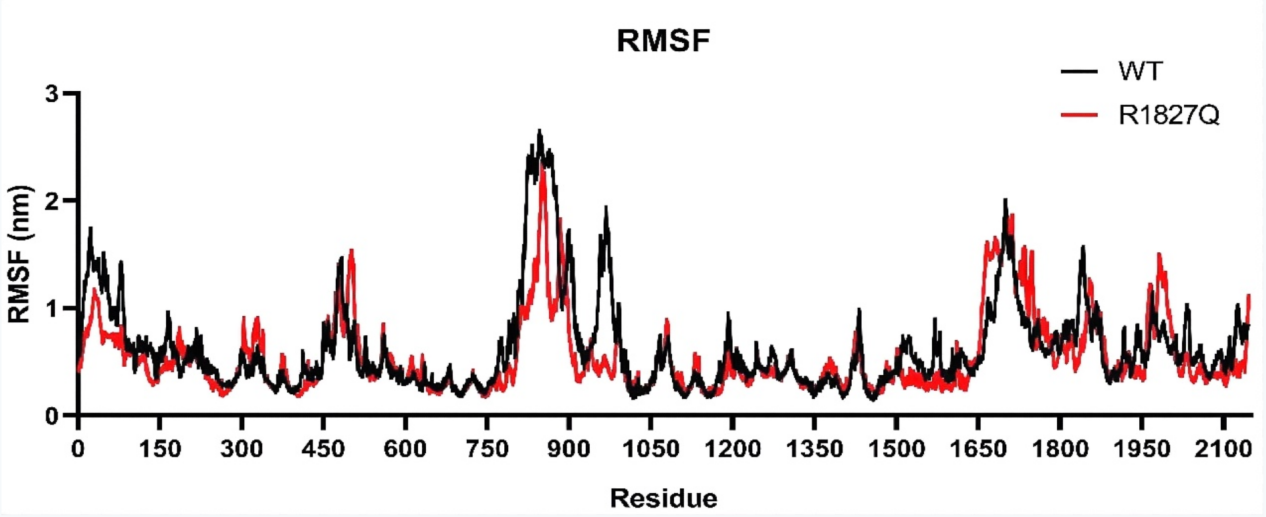


**Fig. S2.** Comparison of *CACNA1C* RMSF before and after *CACNA1C* p.R1827Q.


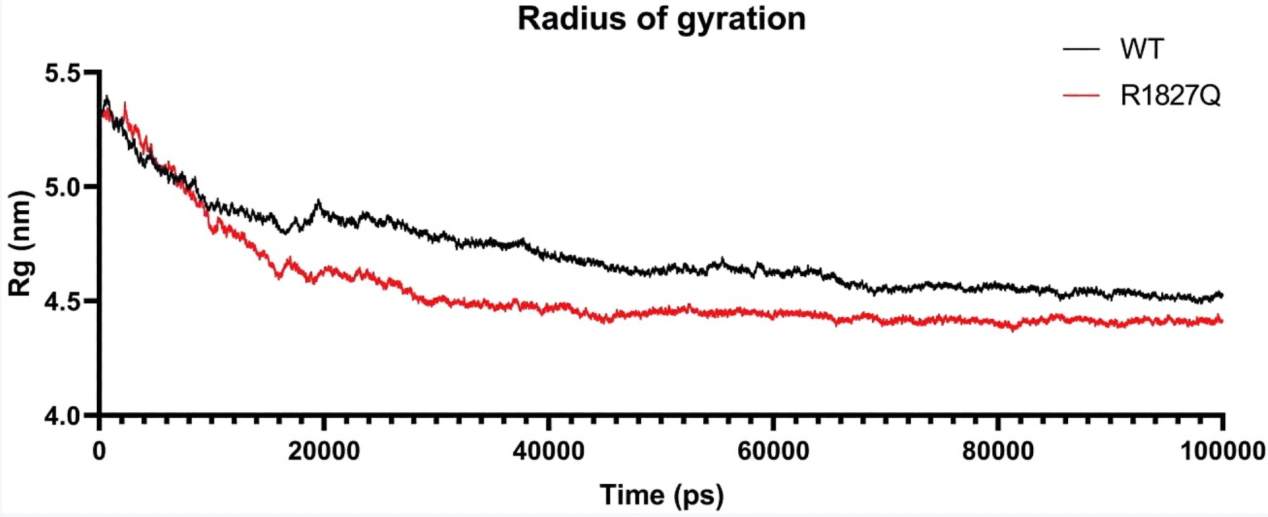


**Fig. S3.** Comparison of *CACNA1C* Gyrate before and after *CACNA1C* p.R1827Q.


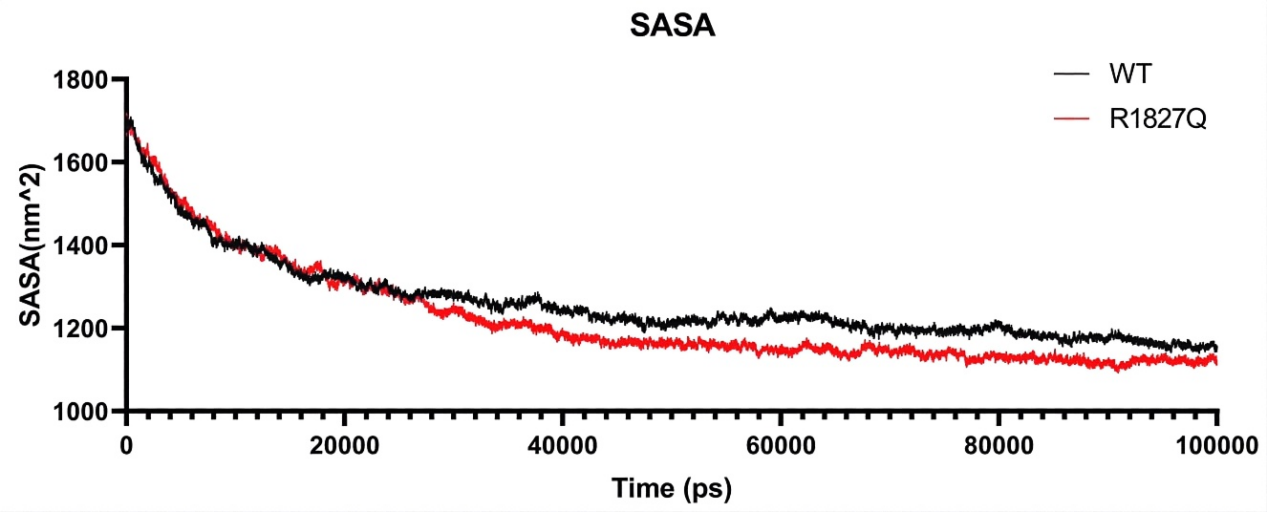


**Fig. S4.** Comparison of *CACNA1C* SASA before and after *CACNA1C* p.R1827Q.


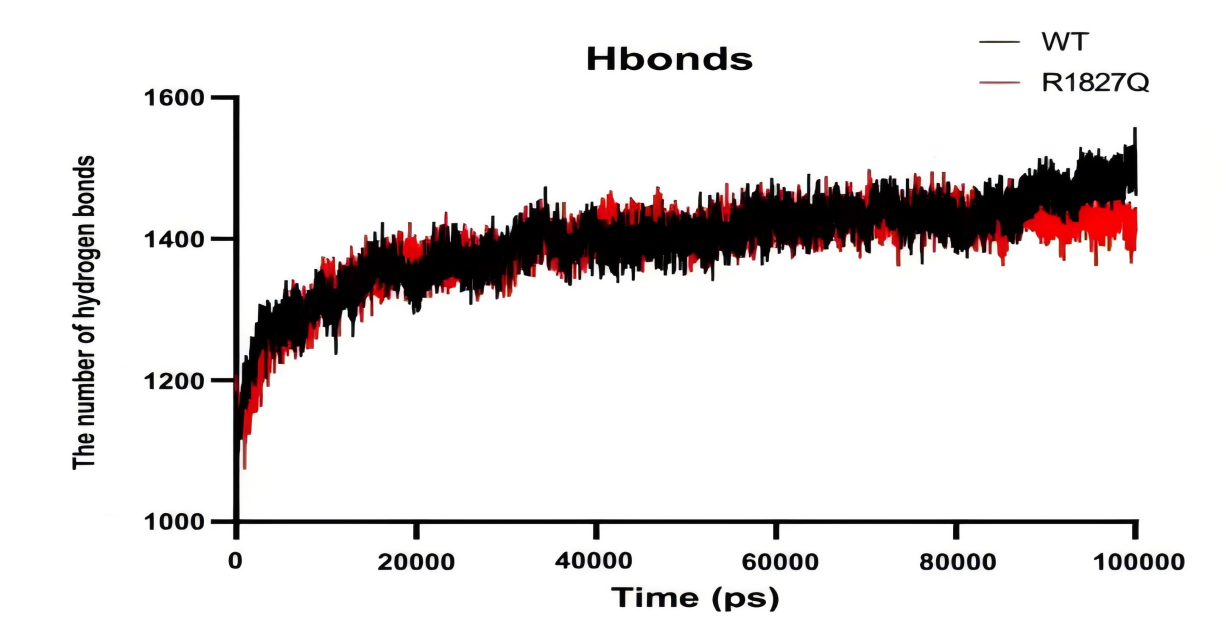


**Fig. S5.** Comparison of *CACNA1C* H-bonds before and after *CACNA1C* p.R1827Q.

# Section 2: Supplementary Tables S1-S3

# **Table S1. Potential candidate genes.**

| **Member** | **Gene name** | **Chromosomal  position** | **Mutation** | **Related diseases** | **Inheritance** |
| --- | --- | --- | --- | --- | --- |
| Ⅲ-31 | CPT2 | Chr1:53676401 | NM_000098  c.1055C＞G  p. F352C | Encephalopathy, acute, infection-induced, 4 | AD/AR |
| Ⅲ-31 | CPT2 | Chr1:53676401 | NM_000098  c.G1102A  p.V368I | Encephalopathy, acute, infection-induced, 4 | AD/AR |
| Ⅱ-17, Ⅲ-6, Ⅲ-31 | KCNT1 | Chr12:2791727 | NM_001272003  c.3596C＞T  p. S1199L | Epilepsy, nocturnal frontal lobe, 5 | AD |
| Ⅲ-31 | SCN1B | Chr19:35530138 | NM_001037  c.556C＞T  p. T189M | Generalized epilepsy with febrile seizures plus 1 | AD |

**Table S2. Harmfulness prediction and pathogenicity assessment of *CACNA1C* variants.**

|  | **Result** | **Annotation** | **Range** | **Source** |
| --- | --- | --- | --- | --- |
| D*/A | 3/4 | D*：numbers of algorithms predicted to be damaging  A：all algorithms used in the study | - | - |
| SIFT | T(0.291) | T：Tolerated | 0-1  <0.05: Deleterious  ≥0.05: Tolerated | Genome Institute of Singapore, 2012 |
| DANN | D (0.993) | D：deleterious | -1-1 (a value close to 1 indicates a high damaging) | University of California，Irvine, 2015 |
| CADD | D (21.7) | D：deleterious | ＞15: Deleterious | University of Washington, 2014 |
| FATHMM MKL | D (0.993) | D：deleterious | 0-1  ＞0.5: Deleterious  ＜0.5: neutral | University of Bristol, 2015 |
| ACMG scoring | PP1+PP3 | PP：pathogenicity supporting. | - | - |
| ACMG  Pathogenicity | Uncertain  significancec | - | - | - |

**Table S3. Domains of *CACNA1C* (NM_001129837).**

| **Name** | **Interval** | **Description** |
| --- | --- | --- |
| Ion_trans | 123-416 | Ion transport protein; This family contains sodium, potassium and calcium ion channels. This family is 6 transmembrane helices in which the last two helices flank a loop which determines ion selectivity. |
| Ion_trans | 523-764 |  |
| Ion_trans | 899-1176 |  |
| Ion_trans | 1218-1476 |  |
| GPHH | 1485-1538 | Voltage-dependent L-type calcium channel, IQ-associated; GPHH is a sequence motif found in this short domain on voltage-dependent L-type calcium channel proteins in eukaryotes. |
| Ca_chan_IQ | 1548-1641 | Voltage gated calcium channel IQ domain;  Voltage gated calcium channels control cellular calcium entry in response to changes in membrane potential. The isoleucine-glutamine (IQ) motif in the voltage gated calcium channel IQ domain interacts with hydrophobic pockets of Ca2+/calmodulin. |
| CAC1F_C super family | 1661-2096 | Voltage-gated calcium channel subunit alpha, C-term;  CAC1F_C is the C-terminal region of voltage-gated calcium channel subunit alpha in higher eukaryotes. |

# Section 3: Supplementary Methods S1-S3

**Supplementary Methods S1: Analysis of targeted exome sequencing data**

Genomic DNA was isolated from a 300-µl peripheral blood sample utilizing the RelaxGene Blood DNA kit (Tiangen Biotech Co., Ltd., Beijing, China). The concentration and purity of the extracted DNA were assessed using a Nanodrop 2000 spectrophotometer alongside a Qubit® 3.0 fluorometer (Thermo Fisher Scientific, Inc., Waltham, MA, USA). Hybrid capture for the genomic DNA was carried out with the Agilent SureSelect Human All Exon v6 Kit (Agilent Technologies, Inc., Santa Clara, CA, USA). To prepare DNA libraries, magnetic AMPure XP beads (Beckman Coulter, Inc., Brea, CA, USA) were used for purification. Targeted enrichment focused on coding sequences and intron-exon junctions, while quality control of the samples was performed via the Agilent 2100 Bioanalyzer system (Agilent Technologies, Inc.). The purified DNA was stored at -20°C until further sequencing steps.

The samples were sequenced on the Illumina HiSeq X Ten platform (Illumina, Inc., San Diego, CA, USA), ensuring high-quality and high-throughput genomic data. The sequencing data were processed and annotated with a custom-developed analytical pipeline. In detail, raw reads underwent preprocessing to filter out low-quality sequences and remove adaptor contamination. Alignment of the reads was carried out using the Burrows-Wheeler Aligner (BWA) tool (version 0.7.17) under default settings, mapping against the hg19 human genome reference (GRCh37) as described in prior studies. The resulting BAM files were sorted using SAMtools for downstream analysis. Variants, including single nucleotide variants (SNVs) and small insertions or deletions (indels, <50 bp), were identified with the Genome Analysis Toolkit (GATK; https://software.broadinstitute.org/gatk). Copy number variations (CNVs) were detected using CNVkit. Furthermore, SNVs, indels, and CNVs were annotated using the Variant Effect Predictor.To assess potential impacts on protein function, two widely recognized prediction tools were utilized: Sorting Intolerant From Tolerant (SIFT) and Polymorphism Phenotyping v2 (PolyPhen-2). For calculating homozygosity ratios based on SNV data, the following criteria were applied: i) SNPs with a sequencing depth below 20x were excluded; ii) only SNPs with variant allele frequencies ranging from 0.2 to 0.8 were classified as heterozygous.

The samples were sequenced on the Illumina HiSeg X Ten platform (Illumina, Inc., San Diego, CA, USA) using the 2×150 bp mode, ensuring high-quality (Q30 > 85%) and high-throughput (average sequencing depth > 100X) exome sequencing data.

**Supplementary Methods S2:** **Sanger sequencing**

Using Primer 5 software, specific primers targeting the upstream and downstream regions of the mutations were designed based on variant sites predicted through whole-exome sequencing. The primers were synthesized by Sangon Bioengineering (Shanghai, China). Genomic DNA amplification was performed using Taq DNA polymerase (TAKARA) with the designed primers serving as templates. After purifying the amplification products, two-directional sequencing was carried out on an ABI 3730XL sequencer. Chromas software was employed to interpret the sequencing data, which were then compared to wild-type sequences to determine their genetic variations.

**Supplementary Methods S3: The specific steps of molecular dynamics simulation**

Molecular dynamics simulations were performed using Gromacs 2022.3 software. Relevant potential data were incorporated into the topology file of the molecular dynamics system. The simulations were conducted under controlled conditions, maintaining a constant temperature of 300 K and a pressure of 1 bar. The Amber99sb-ildn force field was applied, with water molecules modeled using the Tip3p water model as the solvent. To neutralize the total charge of the system, an appropriate number of Na+ ions were introduced. To prepare the system, energy minimization was carried out using the steepest descent algorithm. This was followed by equilibration under isothermal-isochoric (NVT) and isothermal-isobaric (NPT) ensembles for 100,000 steps each, with a coupling constant of 0.1 ps and a total duration of 100 ps. Subsequently, a production molecular dynamics simulation was conducted, comprising 5,000,000 steps with a time step of 2 fs, resulting in a total simulation time of 100 ns. After completing the simulations, the software’s integrated analysis tools were used to examine the trajectory data. Key metrics such as root mean square deviation (RMSD), root mean square fluctuation (RMSF), radius of gyration (Rg), solvent-accessible surface area (SASA), and hydrogen bond (H-bond) formation were calculated. These analyses aimed to investigate structural changes in the protein caused by mutations and to elucidate the dynamic processes involved in protein-protein interactions.

RMSD is a widely utilized metric for evaluating the precision of molecular simulations or protein structure predictions. It measures the positional differences by calculating the distance between each atom in the predicted structure and its corresponding atom in the reference structure. Subsequently, the mean of the squared differences is computed, followed by extracting its square root. A lower RMSD value reflects greater protein stability, whereas a higher RMSD suggests significant structural conformational changes in the protein's backbone during the simulation period.

RMSF is an indicator used for analyzing the dynamic properties of molecular simulations or protein structures. It calculates the root mean square displacement of each atom in the protein and evaluates their fluctuations during the simulation process. The RMSF of a protein is considered to determine the deviation of each residue from the reference position. This parameter determines the applicability of ligand-protein interactions over simulation time. Amino acids far from the active site will show higher RMSF values. Also, RMSF is related to protein flexibility, the more flexible the area, the greater the RMSF value.

The radius of gyration serves as a key metric for assessing the structural compactness of protein molecules. A smaller radius of gyration reflects a higher degree of molecular compactness, offering valuable insights into the spatial arrangement and density of the protein's structure.

SASA is a widely employed parameter to quantify the portion of a protein molecule's surface that interacts with the surrounding solvent. This measure estimates the accessible surface area of individual atoms within the protein and analyzes their contribution to molecular interactions and structural stability. A reduced SASA value suggests diminished exposure of the protein's surface to the solvent, reflecting a more compact structure.

H-bonds represent a specific type of molecular interaction that arises from the attraction between a hydrogen atom and electronegative atoms. These interactions play a crucial role in evaluating intra-protein and protein-ligand interactions, offering insights into their influence on the protein's structural stability and functional mechanisms.
